# The role of RhoA/Rho kinase pathway in endothelial dysfunction

**DOI:** 10.4103/0975-3583.74258

**Published:** 2010

**Authors:** Lin Yao, Maritza J. Romero, Haroldo A. Toque, Guang Yang, Ruth B Caldwell, R. William Caldwell

**Affiliations:** *Department of Pharmacology and Toxicology, Medical College of Georgia, Augusta, GA, USA*; 1 *Department of Vascular Biology Center, Medical College of Georgia, Augusta, GA, USA*

**Keywords:** Endothelial dysfunction, Rho kinase, nitric oxide, endothelium-dependent contractions

## Abstract

Endothelial dysfunction is a key event in the development of vascular disease, and it precedes clinically obvious vascular pathology. Abnormal activation of the RhoA/Rho kinase (ROCK) pathway has been found to elevate vascular tone through unbalancing the production of vasodilating and vasoconstricting substances. Inhibition of the RhoA/ROCK pathway can prevent endothelial dysfunction in a variety of pathological conditions. This review, based on recent molecular, cellular, and animal studies, focuses on the current understanding of the ROCK pathway and its roles in endothelial dysfunction.

## INTRODUCTION

Vascular disease, particularly atherosclerosis is a major cause of disability and death in patients with diabetes mellitus. The pathophysiology of vascular disease in diabetes involves abnormal function of the vascular endothelial and smooth muscle cells (SMC) as well as platelets. Endothelial dysfunction may be a critical and initiating factor in the development of diabetic vascular disease.[1,2] The broad definition of endothelial dysfunction, a systemic pathological state of the endothelium (the inner lining of the blood vessels), is an imbalance between endothelium-derived relaxing factors (EDRF) e.g. nitric oxide (NO), and prostacyclin and endothelium-derived constricting factors (EDCF) e.g. thromboxane A2 (TxA2), prostaglandin H2 (PGH2), endothelin-1 and angiotensin II.[3]

The small GTPase RhoA and its downstream target Rho kinase (ROCK) regulate cellular adherence, migration, and proliferation through control of the actin–cytoskeletal assembly and cell contraction.[4] Since their discovery in 1996, ROCKs have been extensively studied. Much of the work has focused on the role of the RhoA/ROCK pathway in endothelial function. For example, among Rho GTPase family members, RhoA is noted as having a critical role for T cell transendothelial migration.[5] The proinflammatory lipid mediator, lysophosphatidic acid (LPA), has been reported to activate ROCK, p38, JNK, and NF-kappa β pathways in human endothelial cells (EC).[6] Inhibition of ROCK can prevent thrombin-induced intercellular adhesion molecule 1 (ICAM-1) expression and can further inhibit nuclear factor (NF)-kappa β activity[7] and tissue factor expression in EC, indicating that the RhoA/ROCK pathway is involved in the mechanism of thrombus formation.[8] Also, RhoA/ROCK activation by C-reactive protein has been reported to enhance endothelial plasminogen activator inhibitor-1 expression, which may result in atherothrombogenesis.[9] Basal Rho kinase activity is essential for the regulation of endothelial barrier integrity.[10] However, overactivation of RhoA/ROCK by disturbed flow can induce phosphorylation of LIM kinase 2 and cytoskeletal rearrangement, resulting in barrier dysfunction in vascular EC.[11]

RhoA/ROCK is also involved in endothelial NO synthase (eNOS) function, as their activation decreases eNOS expression by reducing the eNOS mRNA stability.[12] Also, use of a ROCK inhibitor can reduce vasoconstriction caused by acetylcholine (Ach) in vessels with an impaired endothelium.[13] Inhibition of the RhoA/ROCK pathway may have significant clinical implications. In this review, we describe the current understanding of ROCK signaling and its role in vascular endothelial dysfunction.

## STRUCTURE, DISTRIBUTION, EXPRESSION, AND FUNCTION OF ROCK ISOFORMS

ROCK is a serine/threonine kinase with a molecular mass of ~160 kDa, which has been identified as the first downstream target of the small GTP-binding protein RhoA.[14,15] ROCK mediates RhoA-induced actin–cytoskeletal changes through phosphorylating the regulatory myosin-binding subunit (MBS) of the myosin light chain (MLC) phosphatase. Phosphorylated MBS inhibits the activity of MLC phosphatase and, thereby, promotes MLC phosphorylation and actomyosin contractility.[16–18]

Two ROCK isoforms have been identified in the mammalian system. ROCK1 (ROKβ or p160ROCK) is located on chromosome 18 and encodes a 1354-amino acid protein.[19,20] ROCK2 (ROKα or Rho-kinase) is located on chromosome 12 and contains 1388 amino acids.[14,21,22] ROCK1 and ROCK2 share an overall 65% homology in amino acid sequence and 92% homology in their kinase domains.[23]

ROCK1 and ROCK2 are ubiquitously expressed in murine tissues from early embryonic development to adulthood. ROCK1 is widely and highly expressed in most tissues except in the brain and muscle, whereas ROCK2 is most highly expressed in muscle, brain, heart, lung, and placenta tissues.[20,22,24] Both ROCK1 and ROCK2 are expressed in vascular EC and SMC.[24–27] Relatively few studies have addressed the regulation of ROCK isoform expression. Angiotensin II (via type 1 receptor) and IL-1 beta upregulate both isoforms of ROCK at the mRNA and protein level in human coronary vascular SMCs. This is mediated by protein kinase C and NF-kappa β.[28] Compensation of ROCK1 for the loss of ROCK2 has not been reported in the ROCK2-deficient mouse.[29] However, in vascular SMC, silencing of either ROCK isoform leads to an increased protein expression of the other isoform, suggesting that the expression level of the ROCK isoforms is tightly controlled and interrelated.[30]

Although ROCK1 and ROCK2 are ubiquitously expressed and highly homologous, several mechanisms have been reported that differentially regulate ROCK isoform activities. For example, the overexpression of ROCK1 and ROCK2 can both increase MLC phosphorylation, but through different mechanisms.[31] ROCK2, but not ROCK1, binds directly to the MBS of MLC phosphatase and plays a predominant role in vascular SMC contractility.[32] ROCK2 is the dominant isoform driving LPA-mediated activation of NF-kappa β and ensuing transcriptional upregulation of ICAM-1 and vascular cell adhesion molecule-1 mRNA and protein in human umbilical vein EC.[33] However, ROCK1, but not ROCK2, knockout mice have a substantially reduced vascular inflammation and neointima formation after flow cessation-induced vascular injury in the ligated carotid artery.[34]

## ENDOTHELIUM-DEPENDENT RELAXATION AND RHOA/ROCK PATHWAY

### Bioavailability of nitric oxide and RhoA/Rho kinase

A hallmark of endothelial dysfunction is reduced bioavailability of NO, which may be caused by reduced expression of eNOS, impairment of eNOS activation, or inactivation of NO by oxidative stress. Accumulating evidence indicates that the expression and activity of eNOS is regulated by the RhoA/ROCK pathway. For example, activation of the RhoA/ROCK pathway significantly inhibits endothelial NO synthase expression and phosphorylation (Ser1177) in the mesenteric arteries of hypertensive profilin1 transgenic mice.[35] Thrombin is reported to decrease the eNOS mRNA level by shortening the half-life of eNOS mRNA via activation of RhoA and ROCK in human EC.[36] Consequently, ROCK inhibitors or statins, which inhibit RhoA activity, can increase the eNOS mRNA half-life and upregulate eNOS expression in animal and human vascular disease. The ROCK inhibitor Y-27632 increased normoxia-induced NO production in the pulmonary artery of late-gestation ovine fetuses infused with nitro-L-arginine.[37] Prolongation of eNOS mRNA half-life by statins is reversed by geranylgeranyl pyrophosphate, which causes the isoprenylation and activation of RhoA GTPase.[12]

### Regulation of signal transduction

There are many signaling molecules involved in the pathogenesis of endothelial dysfunction via impairment of NO bioavailability. Some of the signaling molecules have been reported to have a link with the RhoA/ROCK pathway. These are phosphoinositide 3-kinase (PI3K)/Akt, reactive oxygen species (ROS), and arginase.

### PI3K/Akt and RhoA/ROCK pathway

Akt (protein kinase B) is a serine/threonine protein kinase, which is the key downstream effector of PI3K. PI3K-dependent Akt activation can be regulated through the tumor suppressor phosphatase and tensin homolog (PTEN), which works essentially as the opposite of PI3K. Akt can directly phosphorylate eNOS on serine 1179 (based on the bovine eNOS sequence and equivalent to human eNOS-serine 1177) and activate the enzyme, leading to NO production.[38] Studies have shown that a crosstalk between RhoA–ROCK and Akt regulates eNOS phosphorylation independent of the RhoA/ROCK actions on the downregulation of eNOS expression. The active RhoA/ROCK pathway not only regulates eNOS gene expression but also inhibits eNOS phosphorylation at Ser-1177 and cellular NO production via suppression of Akt activation in human umbilical vein EC.[39] Furthermore, inhibition of RhoA or ROCK isoforms leads to the rapid activation of the PI3K/Akt pathway and phosphorylation of eNOS.[39,40] RhoA and ROCK can directly phosphorylate and activate PTEN,[41,42] suggesting that PTEN may be also involved in NO regulation via RhoA/ROCK and PI3K/Akt complex.

### ROS, arginase, and RhoA/ROCK pathway

It is well known that ROS reduce the bioavailability of NO. The reaction between superoxide and NO forms peroxynitrite, which oxidizes and decreases the level of tetrahydrobiopterin (BH4), a cofactor required for eNOS activity and NO synthesis. Moreover, increased peroxynitrite positively correlates with a significant upregulation of the active RhoA in models of experimental diabetes.[43] RhoA plays a significant role in endothelial permeability, EC migration, and angiogenesis.[44,45] One of the Rho guanosine nucleotide exchange factors (Rho GEF), p115-Rho GEF, is reportedly involved in mediating thrombin-induced pulmonary EC dysfunction,[46] and ROS have been shown to induce vascular contraction through activation of Rho/Rho kinase.[47] Our previous studies demonstrated that peroxynitrite can suppress eNOS expression via activation of RhoA and hence can cause vascular dysfunction.[48]

In addition, elevated arginase activity also limits NO availability. Arginase is a hydrolytic enzyme that converts L-arginine into urea and ornithine. Thus, enhanced arginase activity can decrease the tissue and cellular L-arginine availability to eNOS,[49] which leads to a decrease in NO production and increased superoxide generation due to uncoupled eNOS.[43,50] Arginase-induced endothelial dysfunction initiates a feed-forward cycle of diminished NO levels and further oxidative stress.[43] Our lab previously showed that diabetes and high glucose increase the activity of arginase through enhanced RhoA/ROCK function.[43] Significantly greater RhoA and arginase activity has also been observed in inflammatory bowel disease and TNF-α/lipopolysaccharide-activated human EC.[26] Elevated arginase activity/expression is blocked by the inhibition of RhoA or ROCK, suggesting that activation of the RhoA/ROCK pathway is a critical step toward elevated arginase activity and expression in the vasculature.[26,27,43]

## ENDOTHELIUM-DEPENDENT CONTRACTIONS AND RHOA/ROCK PATHWAY

In vascular diseases, endothelial dysfunction is also due in part to the release of EDCF, which counteracts the vasodilator action of NO or PGI2. The vessel contraction mediated by EDCF is widely called endothelium-dependent contraction in the scientific literature. Although this term is somewhat imprecise, it has become widely used.

In blood vessels, endothelium-dependent contraction to Ach is not observed under normal physiological conditions. However, it is observed under pathological conditions, such as in hypertension and in diabetes, in which endothelial function is markedly impaired.[51–54] TxA2 and/or PGH2, synthesized by cyclooxygenase (COX), mediate endothelium-dependent contraction[55–60] by activating thromboxane–prostanoid (TP) receptors on vascular SMCs.[53,61,62] One of the signaling molecules activated by TP receptor in smooth muscle is Rho kinase.[63] Activated RhoA and ROCK result in the inhibition of MLC phosphatase, which decreases the dephosphorylation of the regulatory MLC. The altered balance of MLC induces contraction of the vascular smooth muscle (VSM) layer.[64]

In the presence of a pathologic vascular endothelial layer, EDCFs may prevail over EDRFs, subsequently inducing activation of the RhoA/ROCK pathway in the VSM layer, resulting in enhanced vasocontractile activity. In clinical studies, the intraarterial infusion of the ROCK inhibitor fasudil lowers blood pressure, and this decrement is higher in hypertensive patients than in normotensive subjects, suggesting that ROCK contributes to endothelium-dependent contraction to Ach.[65] In carotid arteries of spontaneously hypertensive rats (SHR), inhibition of ROCK caused a dose-dependent reduction in the endothelium-dependent contraction to Ach.[13] Also, the contractions induced in the aorta of SHR and Wistar Kyoto rats by Ach can be abolished by inhibitors of ROCK, either Y27632 or HA1077 (fasudil).[13] Furthermore, RhoA–ROCK has been reported to mediate the enhanced endoperoxide-dependent vascular contraction characteristic of hypertension.[13] These findings suggest that inhibition of ROCK can reduce the EDCF-mediated responses and consequently contribute to the lowering of arterial blood pressure in vascular disease.

## CURRENT STRATEGIES FOR STUDYING ROCKS

### RhoA/Rho kinase inhibitors

Although an increasing number of reports show that ROCK plays an important role in endothelial dysfunction, more insights into the molecular mechanisms that contribute to increased ROCK activity or the downstream targets for ROCK are needed. Determination of the precise role of the two ROCK isoforms is limited by the lack of specific and selective pharmacological inhibitors currently available. Statins are indirect inhibitors of the RhoA/ROCK pathway, which act by decreasing the synthesis of isoprenoids. An intravenous injection of pravastatin prevents impaired NO-dependent vasodilation by blocking the full activation of unprocessed RhoA and Rac1 and the downregulation of Akt/eNOS pathways in Wistar and SHR.[8,66] Fasudil was the first ROCK inhibitor approved for clinical use, which inhibits ROCK by competing with ATP for binding to the kinase,[67,68] but it also inhibits other kinases. Hydroxyfasudil (HA-1100), which is an active metabolite, is highly selective for ROCKs. When compared with protein kinase A, the IC50 value is approximately five-fold lower for fasudil and 50-fold lower for hydroxufasudil.[23] Y27632 is another nonspecific inhibitor of both ROCK isoforms by competing with ATP for binding the kinase.[69] At higher concentrations, it can also inhibit Rho-dependent kinase C and A.[68] Recently, two novel compounds, GSK2699624 and SB772077B, were reported to have higher potency than either Y27632 or fasudil, especially in inhibiting ROCK1.[70] More highly specific ROCK-2 inhibitors, such as SR-715 and SR-899, have also been developed.[71] More interest within the pharmaceutical industry will accelerate the development of selective ROCK inhibitors.

## ROCK KNOCKOUT ANIMALS

There is no doubt that ROCK1 and ROCK2 knockout mice are the most accurate and specific way to investigate the in vivo distribution/function of ROCK isoforms. Complete loss of ROCK1 in mice results in the eyelids being open at birth and an intestinal protrusion phenotype,[72] whereas loss of ROCK2 results in placental dysfunction leading to intrauterine growth retardation and about 90% fetal death.[29] However, both groups of haploinsufficient ROCK mice develop normally and are fertile. Indeed, developing studies with ROCK-deficient mice would have the greatest chance of increasing our understanding of the function of specific ROCK isoforms in various diseases. Better yet, development of conditional knockouts for ROCK would be of great value.

## CONCLUSIONS

**Figure 1:**
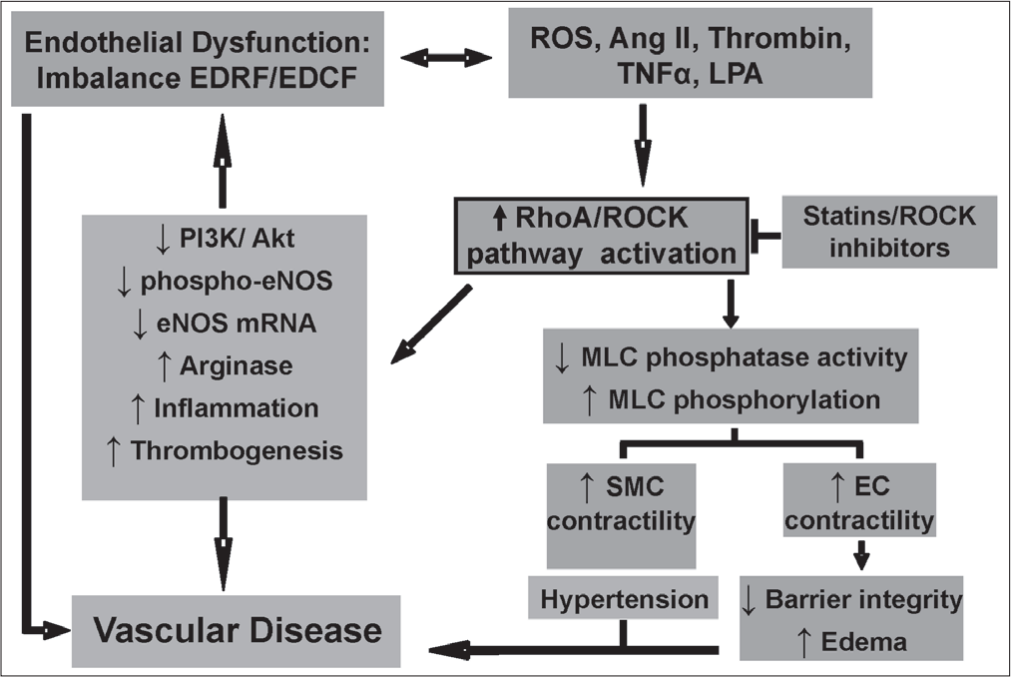
Sustained vascular endothelial dysfunction, defi ned as an imbalance between endothelium-derived relaxing factors (EDRF) and endothelium-derived constricting factors, is induced by various factors (reactive oxygen species, ang II, thrombin, TNFα, lysophosphatidic acid), which lead to vascular disease. The actions of these vascular insult factors observed in diabetes and many other vascular diseases involve abnormal function of endothelial cells and smooth muscle cells (SMC) with altered vascular contraction through RhoA/ Rho kinase (ROCK) pathway activation. This ultimately leads to endothelial barrier dysfunction/edema and enhanced SMC contractility and hypertension. In addition, the RhoA/ROCK pathway plays a central role in impaired production of the EDRF nitric oxide due to multiple actions on constitutive endothelial NO synthase (eNOS). This occurs by reducing PI3K/Akt activation and subsequent reduction of eNOS phosphorylation and downregulation of eNOS mRNA stability. Additionally, activation of the RhoA/ROCK pathway causes elevation of arginase activity/expression, which results in limited availability of the substrate L-arginine for eNOS function. RhoA/ROCK pathway has also been also associated with the mechanism of thrombus formation and vascular infl ammation

There is growing evidence that the RhoA/ROCK pathway has an important pathophysiological role in vascular endothelial dysfunction. Inhibition of ROCK may be an attractive therapeutic target for preventing endothelial dysfunction [Figure 1]. However, a better understanding of the physiological role of each ROCK isoform in the cardiovascular system is needed, and can be resolved by the development of isoform-specific inhibitors and extensive use of ROCK-deficient mice.
